# Driving phronesis as a milestone for engineering autonomous vehicles

**DOI:** 10.1007/s00146-026-03250-z

**Published:** 2026-08-27

**Authors:** Toni Gibea, Radu Uszkai

**Affiliations:** 1 Department of Philosophy and Social Sciences, Bucharest University of Economic Studies, Bucharest, Romania; 2 Research Centre in Applied Ethics, University of Bucharest, Bucharest, Romania

**Keywords:** Driving phronesis, Autonomous vehicles, Digital duplicates, Virtue ethics

## Abstract

One of the main ethical reasons why autonomous vehicles (AVs) have been portrayed as the future of transportation is their potential to enhance road safety by eliminating human error. However, while AVs may mitigate known hazards, their successful deployment in the near future hinges on their performance in a mixed-traffic environment, where they must coexist and interact with human drivers. This paper adopts a broad Aristotelian virtue ethics framework to introduce the concept of driving phronesis as a normative framework for resolving nuanced, everyday coordination problems in traffic. Thus, our paper argues that a promising future for AVs is one in which they will function as digital duplicates of drivers in specific contexts, endowed with functional driving phronesis.

## Introduction

Autonomous vehicles (AVs) are automobiles with a high level of automation capable of independent driving in all scenarios, regardless of the presence of a driver or passenger. In the academic literature, AVs are referred to as robo-drivers, self-driving cars, automated cars, driverless cars, automated driving systems, or highly automated systems, depending on their different levels of automation defined by the Society of Automotive Engineers.^1^ Researchers have also introduced broader concepts such as connected and automated vehicles (CAVs) which capture not only AVs themselves, but the entire digital infrastructure, data gathering, and analysis that support them (Fossa 2023, 2025; Santoni de Sio 2021).

Leaving aside the possible future scenario where we have fully autonomous vehicles, the ethical challenges automated vehicles pose to date lie in minimizing harm while ensuring that the technology serves the social good and enhances drivers’ autonomy (Santoni de Sio and Fossa 2023). This justifies the need for a brand new field of the ethics of transportation (Santoni de Sio 2021).

This paper starts from the presupposition that the ethical decision-making capacities of future AVs should not be treated as a problem for the future. Although fully autonomous agents do not exist yet, companies are actively developing the necessary infrastructure to achieve this goal.^2^ For this reason, there is a strong case to be made to address ethical decision-making by AVs because, as in the case of human development, instilling moral values is a long process that must begin at the earliest stages of development. This would be both easier and more effective than the alternative of simply adding a module or an update for ethics later.

Through a comprehensive literature review, we first identify and analyze existing research on the ethical challenges of AVs deployment and highlight an important existing gap: hybrid decision-making methods would benefit from an explicit integration of key elements from virtue ethics in order to solve complex coordination problems in day-to-day traffic scenarios. To address this gap, we explore the implication of adopting a virtue ethics framework in the case of designing AVs. More specifically, we introduce the concept of *driving phronesis* as a normative framework for resolving nuanced, everyday ethical conflicts in traffic. We tentatively define driving phronesis as the ability to be action oriented, to make driving decisions which are context-dependent and based on knowledge acquired through experience, looking especially at complex driving coordination situations.

The need for such a framework becomes especially pressing in a complex mixed-traffic environment, where AVs must coexist and interact with human drivers (Nyholm and Smids 2020). Thus, our paper will end with a blueprint for instilling what we label as *functional* driving phronesis in AVs supplemented with a couple of case studies aimed at making the case for our approach. We believe that our strategy introduces a distinct set of challenges which require, but cannot be reducible to, technical solutions.

## Ethical bumps on the road for AVs

Ethicists have long been concerned with how AVs should make decisions when facing a moral dilemma, with many believing that theoretical trolley problems would directly guide us in solving real-life ethical dilemmas. Experts point out that even in highly implausible scenarios in which automated cars are technically flawless, equipped with perfect sensors and mapping, an AV must still be capable of making a decision if, for example, a pedestrian falls directly in its path (Goodall 2014; Patrick 2015). Should an AV risk their passenger’s life by steering into the opposite lane to save a vulnerable pedestrian?^3^ How should it choose between risking the life of one passenger or the lives of five pedestrians?

There are two well-known normative theoretical approaches within which philosophers try to answer questions related to sacrificial dilemmas (e.g., is it permissible to sacrifice one life to save five?). A Kantian-inspired theory might prioritize the Principle of Humanity, treating a person never as a means, but always and at the same time as an end in itself. Conversely, a simple act utilitarian might argue that if a decision saves more lives, it should be made regardless of other factors. Interestingly, both moral theories could be implemented technically, with cost functions in the case of utilitarianism, and side constraints for actions in the case of deontology, but certain cases that show how much friction there is between these two theories cannot be ignored (Gerdes and Thornton 2016). In what follows, we argue that the philosophical literature on trolley-like dilemmas (Kamm 2015) is not directly relevant to specific AV dilemmas. We will also highlight the existing hybrid solutions advanced for AV moral decision-making methods, focusing on existing coordination problems in traffic.

### From theory to practice

In his 2018 paper, Himmelreich offers an in depth analysis of why we should approach trolley dilemmas with caution when discussing the ethics of AVs. He lists a series of issues, ranging from the fact that trolley cases are inconsistent to the fact that they only allow a top-down design approach.

Another problem is that trolley cases treat design issues as a purely moral problem, ignoring the need for a political solution. Focusing exclusively on theoretical potential dilemmas obscures the fact that we need political consensus on the legal aspects surrounding AVs which will be deployed in real traffic environments. When doing conceptual work (e.g., testing the doctrine of double effect), philosophers can do away with criminal law. More precisely, they are not interested in the legal consequences someone would face for pushing a person with a huge backpack in front of a runaway trolley. But when we discuss the way in which an AV should drive on the roads of San Francisco or Guangzhou, legal consequences should not be ignored. It would be reckless to deploy disruptive technologies before adapting the existing legal framework to new situations.

Furthermore, trolley cases open up a can of epistemic worms due to real-life uncertainties. For example, in footbridge dilemma situations, philosophers just assume that someone with a huge backpack will stop the trolley, if pushed in front of it. Questions about the feasibility of such a scenario and outcome could derail philosophers from their theoretical objectives. An AV should be equipped with the ability to recognize whether it faces a sacrificial dilemma based on real-life probabilities. Just recognizing that a child is alone on the sidewalk should not trigger the cars to drive onto other cars because there is a probability the child could start running in front of the car. Or, even a better example, try to save 5 people by getting in front of a heavy-duty commercial truck, fully loaded, because there is a slight chance that your small car would stop it. Imagining dilemmas is useful for theoretical purposes, especially when one explores concepts, notions, definitions or principles, but not when the purpose is to deploy a functional self-driving car.

In contrast with trolley cases, Himmelreich argues for the necessity of studying real traffic scenarios which have a more mundane feel, “such as approaching a crosswalk with limited visibility, making a left turn with oncoming traffic, and navigating through busy intersections” (2018, p. 678). These also raise relevant concerns for designing AVs and they make room for important theoretical normative discussions.

### Theoretical adjustments for AV road conditions

Poszler et al. (2023) map the advantages and disadvantages of designing a specific moral decision-making method from a specific moral theoretical perspective. They list six potential theories (deontology, virtue ethics,^4^ consequentialism/utilitarianism, contractualism, risk ethics and descriptive ethics) that could inspire a method of decision-making.

Deontology offers a clear ruled-based method. It provides a more reliable blueprint for predictive behavior of AVs. It might also be useful in addressing some important legal conundrums, like liability in unavoidable collision scenarios, by embedding rules and laws in AVs by design (Pickering and D’Souza 2023). There is, however, a clear drawback of a deontological approach: what will those rules look like? Demanding a list of specific rules, or commands, and a clear method of deriving these commands from other more general rules or principles is a substantial challenge even for moral philosophers that have no regard for the field of applied ethics.

Utilitarianism (or any consequentialist approach for that matter) comes with the advantage of being more flexible than Kantianism. It takes into account more factors and manages to avoid stand-still scenarios in which duties or norms conflict with each other (Thornton et al. 2017). As expected, utilitarian or consequentialist methods aim at maximizing the greatest good in traffic scenarios (Kumfer and Burgess 2015). While it might sound easy on paper, this is no simple task. What sort of felicific calculus should we employ in traffic conditions? Can we ignore unfair decisions made by AVs in traffic for the greater good? While this is clearer in cases involving sacrificial dilemmas, it is still relevant for other types of mundane traffic interactions like speeding or running a red light when we could maximize the greater good.

Contractarianism offers another framework not only for designing artificial agents (Leben 2018), but also for evaluating the economic impact of AVs (Dubljević and Bauer 2022). When it comes to how AVs should drive, one proposal for a contractarian algorithm (Leben 2017) draws inspiration from Rawls. It can assist the decision-making process by determining payoffs and choice, based on a Veil of Ignorance: the best option is the one which rational participants would choose in this modified original traffic position. Such an algorithm has more degrees of freedom in assessing multiple, ethically relevant aspects compared to utilitarianism and the double-effect doctrine. For example, for a contractarian-inspired algorithm, it facilitates in practice the decision to change the lane of a moving vehicle and put at risk five occupants to avoid hitting one pedestrian if the maximum risk of the five is a few bruises plus property damage, while the risk for the pedestrian is potentially a few days of hospitalization. This framework could be further decentralized in order to create an environment in the system in which norms could also emerge (Mashayekhi et al. 2022): AVs would be capable of creating entirely new rules by accelerating the processes through which humans usually create norms when interacting with each other.

Because the concept of risk is so vital for autonomous driving, it made some reconsider the role normative theories have in the context of enabling an AV to possess a certain level of decision-making (Goodall 2019, 2016). The issue with the strategy of replacing traditional normative frameworks with the concept of risk is that normative assumptions exist even if we use a concept that is not specific to ethics. For example, Bonnefon (2021, p. 145) talks about how the European Union^5^ regulated car bull bars in order to transfer some of the risk from pedestrians to vehicle occupants. The underlying moral justification for this policy is quite clear: pedestrians are way more vulnerable when involved in car accidents than vehicle occupants. Other derivatives are more honest in the sense that they recognize their ethical assumption and instead of replacing a certain normative framework, expand on the ethics of risks and propose practical solutions. For example, in terms of risks, Geisslinger et al. (2023) developed an algorithm in order to assess the risk of different routes for a vehicle, with probability-based predictions, and distribute the risk fairly among the traffic participants.

Descriptive ethics uses sacrificial ethical dilemmas and their derivatives as tools that can be used to elicit people’s moral intuitions on how an AV should decide and act, measuring such preferences. The Moral Machine experiment collected approximately 40 million decisions from 2.3 million participants across 233 countries and territories. It presented participants with vignette-based scenarios in which an autonomous vehicle had to choose between different sacrificial outcomes, such as saving a human rather than a cat, a child rather than an adult, or a businessperson rather than a person experiencing homelessness. These scenarios also included more traditional trolley-style dilemmas, such as sacrificing one person in order to save five (Awad et al. 2018). Their results showed that moral preferences vary across at least three different clusters: Western, Eastern, and Southern. In other words, AV ethics must confront the tension between cultural moral relativism and moral realism. However, the reliability of the Moral Machine findings has also been questioned. Some researchers argued that traditional survey-based methods fail to capture how people respond to ethical dilemmas in driving situations in practice, and have therefore proposed more immersive methods, such as virtual reality, to study moral decision-making in AV contexts (Wilson et al. 2018, 2019).

### Hybrid decision-making methods for AVs

Since a single normative framework comes with both advantages and disadvantages, some argue that hybrid versions for AV decision-making could offer a solution for addressing individual tradeoffs (Poszler, et al. 2023, 2024). There are multiple categories of hybrid decision-making methods: (a) a mix between traditional normative theories, (b) a mix between risk ethics and traditional normative theories with additional principles and considerations as add-ons, and (c) hybrid theories where traditional normative frameworks are combined with descriptive ethics.

Another proposal for the ethical design of AVs is to implement a three-step procedure which involves (i) collecting relevant moral intuitions, (ii) justifying them through a reliable method which shows both which intuitions are reliable and which reasonably conflict, and (iii) deliberate politically to reach a consensus (Cecchini et al. 2025a, b). This has some similarities to the Delphi method of decision-making (Millar et al. 2007; Muresan 2012; Rauch 1979), but there are also relevant differences. The model is based on data describing common sense morality, which is actively and extensively studied in multiple fields of research. In principle, all the data from the Moral Machine experiment is assessed normatively and filtered. Some intuitions might be normatively reliable, while others might be just arbitrary reactions. Could the empirical data tell us something significant about our normative stand? Without referring to AVs, this line of thought was first articulated by Singer (2005). He is one of the first philosophers who used data from experimental psychology and neuroscience in order to defend utilitarianism. Of course, the data should not be used in order to a priori validate a certain normative framework. Furthermore, we are aware that some of our deontological intuitions are backed up, in traffic scenarios, by self-interest or an instinct of conservation which makes the argument, on the whole, normatively less reliable.

An even more refined alternative would be to limit the concepts advanced by multiple normative frameworks to a few (and more specific ones) which could be easily operationalized. The agent, deed, consequences (ADC) model (Dubljević and Racine 2014) does exactly this. This hybrid method posits that there are, in principle, three classes of normatively significant information. The character and the intentions of the agent (A) are highly relevant when we examine whenever someone behaved morally or not. You can act ethically but for the wrong reasons or in order to achieve a greater moral goal. In what concerns the deed (D), we look at the action isolated from other factors, and then we factor in the consequences of the action (C). Basically, ADC manages to combine virtue ethics, deontology, and utilitarianism and offer a functional way of assessing different scenarios. ADC could also be applied to other mundane types of moral scenarios without focusing exclusively on dilemmas (Cecchini et al. 2025a, b). This framework was also successfully tested to better understand how humans make moral judgments in AV scenarios and normatively assess these judgments (Pflanzer et al. 2025).

The need for all of these hybrid versions proves one thing: complex traffic situations demand a high level of coordination, and there is no easy, straightforward way to fully account for them normatively. In addition to this, AVs could cause harm if they fail to integrate with other traffic participants, with three potential scenarios advanced by Nyholm and Smids (2020): (i) AVs align with human driving behavior, possessing the ability to interpret and even “bend” rules; (ii) humans remain involved in the driving process of highly automated cars^6^; (iii) technology is used to coerce humans into driving like artificial agents.^7^

Taking into account the purpose of our paper, the only relevant scenario advanced by Nyholm and Smids (2020) is the first one: in places like San Francisco, AVs are already deployed in mixed-traffic scenarios, in which they have to coordinate with human drivers in complex traffic situations.

### Traffic coordination problems and the struggle for duplicating drivers

Coordination problems in traffic could be caused by a wide variety of factors: bad infrastructure, bad driving habits, bad judgment, lack of relevant information, and the occurrence of unexpected events. While human drivers could be considered the main failure point of traffic coordination problems, they also have the ability to solve most of them. Some develop better driving skills, like braking late, reacting faster when accelerating or changing direction in order to optimize traffic for themselves and other drivers.

In order to understand the challenges of AV-human driver interactions, one useful example consists in how traffic participants communicate with each other. For example, human drivers use the main lights to flash each other when letting others merge onto their lane. They also use hazard lights as a way of saying “thank you” to other drivers who slow down and allow them to change lanes. Other coordination problems require more knowledge and probably relevant experience. What is more, the overwhelming majority of the situations in which AVs are already involved are, in Himmelreich (2018) words, “mundane” events, not sacrificial dilemmas. Thus, for some software engineers, the solution to coordination problems in traffic consists in aligning AVs with human driving behavior through end-to-end imitation learning via behavioral cloning (Samak et al. 2021).

In what follows, we propose to expand on hybrid ethical decision-making methods by arguing that any hybrid approach of mixed-traffic scenarios for AVs should make room for some important virtue ethics elements. For instance, what the end-to-end imitation learning approach lacks is an ethical framework which takes into account the challenges that this cloning behavior entails and guides software engineers in the process. Moving beyond simply deontological constraints, utilitarian calculus, and moral intuitions, we propose that a milestone for engineering AVs should start from the following questions: can drivers possess a form of phronesis and, if they do, can it be duplicated in artificial agents?

## Digital duplicates, virtue ethics, and functional driving phronesis: a new blueprint for designing AVs

Within the field of the ethics of new and emerging technologies more generally, and AI ethics and machine ethics specifically, normative approaches which draw heavily from virtue ethics have gained momentum in the past decade (Beier 2025). This development has given rise to two important branches of applications. The first one focuses not on the technologies per se, but on the relation that various stakeholders (engineers, designers, users and even society as a whole) have with complex artifacts and on whether these relationships help us live flourishing lives. The key question, here, is what type of virtues do we need when we develop, design, or use new and emerging technologies. The second approach turns to technologies and asks whether we could build machines which display/instantiate human (or human-like) virtuous behavior. This shift from deontology and utilitarianism towards virtues is motivated by the perceived limitations of these previously dominant normative theories, particularly in addressing questions of system design and moral responsibility.

On the one hand, trying to solve complex moral dilemmas raised by the development and deployment of AI systems and artificial semi-autonomous agents by simply embedding various deontological, utilitarian, or contractarian moral principles and rules fails to address others, at least as important issues. For instance, regardless of how we might solve complex responsibility puzzles, to what degree does an increased reliance on such tools (be they conversational chatbots or AVs) lead to deskilling in various areas of our lives, including (in some instances) our moral capacities? For this reason, virtue theorists argue that any roll out of AI technologies needs to be explored within a larger framework, one in which we should take into account the extent to which they actually contribute to human flourishing (Farina et al. 2024). Thus, for some authors like Vallor (2016), technomoral virtues emerge as a solution to tackle the problem of “technosocial opacity” (Vallor 2016, p. 23) which raises serious issues for utilitarians and deontologists. As Vallor points out, adopting a utilitarian perspective leaves open the possibility of sacrificing the well-being of a minority for the greatest overall happiness. Deontology, at least with a Kantian flavor, is equally at odds with the requirements posed by new and emerging technologies, especially since it emphasizes the pivotal goal of rational consistency, while explicitly ignoring important vectors of morality like natural human bonds and caring for each other. In short, utilitarianism and Kantian deontology do not say anything about how technologies could help us live fulfilling lives. In contrast with utilitarianism and deontology, a virtue ethics framework applied in the context of new technologies allows for significantly more flexibility, because it changes our focus when it comes to what sort of moral expertise we actually require and it has the potential to enrich any hybrid decision-making methods. For utilitarians and deontologists, moral expertise is nothing more than an abstract cognitive ability related to abstract rules and principles. For virtue ethicists, it includes much more: “emotional and social intelligence: keen awareness of the motivations, feelings, beliefs, and desires of others; a sensitivity to the morally salient features of particular situations; and a creative knack for devising appropriate practical responses to those situations, especially where they involve novel or dynamically unstable circumstances” (Vallor 2016, p. 26). Thus, in contrast with competing approaches, technomoral virtues avoid problems related to rigidity, reductionism and abstraction from context and allows us to navigate better in conditions of moral novelty and uncertainty.

On the other hand, virtue ethics is also seen as a complement to the dominant principle-based approach of AI ethics in the wake of the latter’s shortcomings (Hagendorff 2020; Mittelstadt 2019). If the crux of the moral problem related to AI ethics revolves around the individuals who are developing and deploying such systems (e.g., engineers, computer scientists or managers), then one important way in which we could ensure compliance with ethical guidelines rests upon cultivating various basic and second-order virtues which would help in making individuals and organizations more virtuous (Constantinescu et al. 2021; Hagendorff 2022).

### Machine virtue ethics for AVs: roadblocks ahead

While not completely ignored in recent debates on drafting decision-making procedures for AVs, virtue ethics is featured more peripherally in the academic literature. A reason for this stems, according to Gurney (2016), from the fact that providing a virtue-based account for what self-driving automobiles should do in the event of a potential crash is not a straightforward task. Nyholm (2018) suggests that it would be useful to integrate virtue ethics within the debate regarding AVs by looking into the broader implications of concepts like virtue, flourishing, and excellence on automated driving in a more general, holistic way. For Nyholm, one key “lesson from a virtue ethical perspective is that we should try to design and program cars in ways that help to make people act carefully and responsibly when they use self‐driving cars.” (2018, pp. 6–7). The focal point of attention, therefore, is not the AV or any artificial moral agent involved in making decisions in sacrificial dilemmas, but on the broader, social and organizational context in which such products are developed, sold, and deployed.

For others, the usefulness of virtue ethics is not confined solely to these broader ethical implications of automating driving, but also to a more detailed agenda (Kulicki et al. 2019; Gerdes 2020; Wapniarski 2025). First of all, applying virtue ethics to AI systems that power AVs is a promising avenue even in sacrificial dilemma scenarios because it gets us closer to moving from “abstract considerations to the actual performance of the system and its improvement” (Wapniarski 2025, p. 1). If there are no perfect solutions to traffic sacrificial dilemmas, then our focus should be on what might make traffic at least incrementally safer in the future, where AVs will have to interact with human drivers.

Furthermore, while acknowledging that one of the main drawbacks of virtue theory in comparison to other ethical frameworks (i.e., deontology, utilitarianism, contractarianism) is the lack of a clear and explicit criteria for guidance in traffic scenarios, Gerdes (2020) strives to show that there is ample room to integrate important technomoral virtues like care, civility, courage, or flexibility in the process of developing AVs. Last, and more importantly, Kulicki et al. (2019) show how AVs themselves could end up espousing important virtues associated with traffic and mobility. Since, as they note, most AVs are based on machine learning applications, learning patterns for what an automobile should do in various traffic conditions (e.g., when to hit the break, accelerate, change the lane, etc.) can be viewed as a form of exercise and habituation which is closely tied with acquiring, in Aristotelian terms, virtue.

In order to briefly evaluate such proposals, we need to take a step back and provide a short introduction to the main tenets of virtue ethics. While it might sound like an oversimplification, we will follow Annas (2011) and Crisp (2018) in identifying the roots of virtue ethics in Aristotle’s *Nicomachean Ethics*. For virtue theorists, the focus is on moral character and how our actions reflect a set of important moral virtues like courage, justice, or prudence. Understood in Aristotelian terms as a disposition of the soul which is acquired through praxis (NE 1105b20-1106a15; 1103a30-b25), virtues require a long process of habituation. During this process of habituation, one intellectual (dianoetic) virtue plays a quintessential part: phronesis*.* Phronesis, also known as practical wisdom, is what “enables agents to make the right deliberations for action relative to the context of their action, to make good sense of variables” (Constantinescu et al. 2021). Thus, in Aristotelian lingo, for an agent to be able to act virtuously, it has to be a phronimos.

The emphasis put on moral deliberation in virtue ethics is at odds with the abilities that artificial moral agents are able to exhibit. For example, Constantinescu and Crisp (2022) distinguish between being virtuous and behaving in a virtuous way. They argue that robotic AI agents would need to meet three criteria in order to be considered virtuous: such agents would have to carry out the right actions, for the right reasons, and in the right circumstances (Constantinescu and Crisp 2022, pp. 1549–1522).

While an AI agent (e.g., an AV) could perform the same external action as a virtuous human being, they lack the essential preconditions to actually act for the right reasons and in the right circumstances. As opposed to a virtuous human being, an AV lacks a moral character developed over a lifetime and the ability to become a phronimos because of the algorithmic and mathematical constraints in which their decisions are made. Simply inferring patterns from a dataset is not the same as understanding context-sensitive details involved in the process of moral decision-making.

One crucial point that we need to take into account here is that a virtue ethics perspective goes beyond an adapted version of Danher’s “ethical behaviorism”. Sure, artificial agents may act in functionally indistinguishable ways to what a human might do. We could go even further and assert that their actions might be “roughly performatively equivalent” (Danaher 2020, 2023)  to those of virtuous humans in various contexts. For virtue theorists, the displayed behavior is just one piece of a more complex puzzle. Focusing on *what* type of behavior an artificial agent displays might say nothing at all about *why* it behaved in that way. This is where the conditions of acting for the right reasons come into full force. What AI agents lack, by design, is an essential precondition: the phenomenological building block required to be able to act for the right reasons. As Constantinescu and Crisp (2022, p. 1550) show, “the fact that the virtuous person acts for the right reasons means that these reasons have been integrated or embedded in their way of being. Virtuous agents phenomenally perceive their situation, including reasons and motivations for acting virtuously”, whereas artificial agents (even those that act in indistinguishable ways from humans) are constrained by their design to infer patterns of behavior based on their algorithmic structure.

Thus, artificial agents are actually at odds with ever being moral agents in the first place (Constantinescu et al. 2022). They fail on account of the control condition, since they do not single handedly cause an outcome only through their own uncoerced will or intention: the boundaries of their world and their functionally indistinguishable ‘choices’ are fleshed out by their algorithmic building block. Without a phenomenological connection to the world, they fail the epistemic condition as well: not being able to act based on the right reasons means not being able to deliberate, one key condition required to be virtuous. Last but not least, without control over one’s action and the capacity to morally deliberate, i.e., if you are not a moral agent, you are unable to develop a moral personality or to genuinely learn from your experience. Therefore, if one lacks genuine moral experience and moral personality, one cannot be considered a genuine moral deliberator who possesses phronesis (Constantinescu et al. 2022).

Analyzed within this framework, proposals like the one from Kulicki et al. (2019) need to be taken with a grain of salt. Due to their inherent ontological and metaphysical properties, AVs cannot be properly considered virtuous (or vicious) regardless of their behavior on the road, but only as behaving in a virtuous way, or as potentially embodying virtue and moral character (Wallach and Vallor 2020). Even though AVs cannot be, in proper Aristotelian terms, virtuous, this does not mean that we should throw the baby out with the bathwater. While not on par with a virtuous human, an AV that embodies virtue would still solve many complex puzzles inherent in driving and transportation. Therefore, what we need to provide is a potential blueprint that engineers need to follow in order for the AVs to reach such desirable goals.

### Functional driving phronesis and the idea of digital duplicates on the road

In most cases, driving on the road rarely involves sacrificial dilemmas. Furthermore, such sacrificial dilemmas lack any sort of satisfactory answers from moral experts and laypersons alike. In fact, most traffic scenarios, while having obvious moral components, are more mundane (see Sect. 3.3 for more details). Therefore, we argue that our focus when designing the algorithmic moral building blocks of future AVs should not be based on answers to idealized Trolley like scenarios, but on how virtuous drivers solve complex coordination problems with other traffic participants (be they other drivers, bicyclists, motorists or pedestrians) in particular settings. Relying extensively on experience and habituation, this is the primordial soup within which, in Aristotelian terms, one would expect virtue to emerge.

While the philosophy of driving is a rather underdeveloped part of the philosophy of transportation, some authors have highlighted that driving, as a human activity, entails the development of virtue by becoming what we could label a *driving phronimos*. In a seminal book in this philosophical niche, Crawford (2020, pp. 121–125) adopts an explicit Aristotelian framework when discussing the skills that drivers acquire and the extent it stops being solely a technical skill, transforming into a process of habituation by the use of practical judgment.

An experienced skilled driver possesses the ability to control a vehicle very well, to stop in a designated space, change lanes properly, avoid other drivers and even mitigate certain risks. But some skilled drivers could also act recklessly because they are overly confident, or they care less about their or others well-being. Both skilled and unskilled drivers could be morally motivated, have a concern for the well-being of others but also a concern for the greater good, but only morally motivated skilled drivers are in general labeled as good drivers.

We argue that there might be another category of skilled morally good drivers, those who are also wise and possess what we call driving phronesis. The actions of good drivers may overlap with the actions of drivers that possess phronesis. For example, both could give priority, although the rules do not tell them to do so, to another car that intends to merge into the main lane. But there are a lot of other situations where being a good driver is not enough, especially when dealing with puzzles created by the infrastructure or by the driving norms themselves. Good drivers respect traffic rules because they deem them as essential, benefiting everyone impartially. Sadly, when such drivers are confronted with conflicts or dilemmas, most of the time, they stick to the same formal traffic rules that do not apply to un-paradigmatic occurrences, while a phronimos at the wheel is able to set good goals and achieve them effectively and realistically without stopping in the middle of the road and debate traffic rules or principles.

Being able to recognize that sometimes reacting faster, even driving more aggressively, might be the norm in a certain town is also essential to understand the particular context in which individuals make decisions at the wheel. In Bucharest, Romania, one needs to react quickly when there is a green light or want to merge onto other lanes. But a driver who possesses phronesis can adapt this strategy. For example, in Thessaloniki, Greece, people have a higher preference for mopeds and scooters as means of transportation. Wise drivers who face for the first time such challenges and lack context-dependent, relevant knowledge, can recognize the fact that they should act in a less aggressive manner in such a traffic setting because, although the streets are crowded and it might be fully justified to react swiftly, one should show more care for more vulnerable motorcyclists. The same thing applies in other countries where merging without waiting in queues is a serious offense.

Anticipation is another criterion that draws a distinction between a good, skilled driver, and a phronimos driver. Being able to read the traffic and anticipate situations, including those where we do not have a clear normative solution, could bring better outcomes, lower the risk and increase efficiency overall.

Possessing the ability of assessing an individual or their character, in Aristotle’s words, increases our capacity of anticipating behavior. While this sort of framing might sound highly problematic for several different reasons, compared to contemporary frameworks which look only at actions without trying to judge the character of the agent, there are also some advantages. In theory, it could guide us as becoming better at predicting which agents are more successful in acting morally. If we assume that both a wise and a good person are equally morally motivated to act in a certain way, different other factors come into play when we look also at the way agents act. In the case of driving, anticipating certain things is definitely a characteristic for a driver who possesses driving phronesis. A wise driver does not just formally understand traffic rules, they understand how they apply in real-life traffic scenarios, understanding the cultural setting and what are their limitations. Therefore, we can boil down the essence of driving phronesis as the ability to answer the following question in particular (including cultural) settings: what is permissible, how, and when.

While the AI system that is the backbone of an AV cannot engage in moral deliberation while in traffic since it cannot be considered a true phronimos, Kulicki et al. (2019), alongside Muntean and Howard (2014), show that there is ample room for training smart algorithms within a virtue ethics framework. Constantinescu and Crisp (2022) also carve out some important ways in which robotic AI systems could be accommodated with virtue ethics. While such agents could not *be* virtuous, they could *display* functional properties related to virtue ethics as a result of their training: “[t]he form of training that AI could undergo to display functional prudence would at most equip it with some form of behavioral competence, but not comprehension” (Constantinescu and Crisp 2022, p. 1552).

In Sect. 3.1, we highlighted the pivotal role played by phronesis in acquiring and practicing virtue. We will now proceed to put forward a proposal that introduces the concept of functional driving phronesis as a normative framework for designing AVs. We define it as the ability of autonomous systems to be action oriented, to make driving decisions which are context-dependent and based on knowledge acquired through experience.

Taking into account the intricacies of the decision-making process involved in driving a vehicle in particular cultural settings and the fact that many of the potential problems that might arise with integrating AVs on the road revolve around their interaction with human drivers, the solution to the gap in the literature that we put forward is to develop AVs as digital duplicates (DD) of idealized versions of drivers in specific parts of the world, which allows for training *functional* driving phronesis. Already a feature in the entertainment industry, DDs harness the power of LLMs and Generative AI in order to create “partial, at least semi-autonomous, digital recreations of real people” (Danaher and Nyholm 2025, p. 2). They could be either visual/auditory or cognitive duplicates and, depending on their deployment, function as digital avatars on screens or embodied robotic avatars in synthetic bodies.

For the purpose of our paper, DDs would provide the blueprint for training AVs in order for such artificial systems to develop functional driving phronesis. Since traffic problems are mostly local problems of coordination, then what we need to allow these systems via their training is to display the functional elements of being a phronimos on the road in Bucharest, Cairo, or New York. This entails providing AVs with the building blocks of an artificial form of phronesis that would emulate the virtues displayed by idealized versions of the median driver on the road in specific places on the planet. Such a functional form of phronesis by design would provide the system with the capacity to emulate right deliberations for actions relative to the driving context of their action, taking into account the type of interaction that the system might have with other traffic participants: not only other drivers, but pedestrian, bicyclists, etc. Therefore, in order for such autonomous systems to gain the capacity to act the right way and based on the right ends, we would need to equip them with driving virtue modules carefully curated for the specific place where the car is driving. Complex traffic coordination problems, as we will see in the next section, can only be solved by having real drivers interacting with DDs of their own who possess functional driving phronesis.

There is one clear risk that our proposal opens up: potentially hard-coding a specific type of driving behavior as being virtuous for the simple reason that it is widespread behavior and used by traffic participants in complex coordination problems. We agree that not all solutions to traffic coordination problems are equally good and not all median drivers should be taken into account in thinking about what behavior we should duplicate on the road. We do not take the “is” of driving as grounding the “ought” of duplicating driving behavior and the design of AVs. One reason is that our proposal focuses on duplicating virtuous driving behavior, therefore we duplicate “oughts”. Of course, these “oughts” are identifiable in practice and we can describe them, but it does not mean automatically that they are reducible just to descriptions. The second reason is that this process of engineering such DDs needs to take into account the feedback of multiple stakeholders involved in transportation systems, including pedestrians and bicyclists. Another, and more important reason, is that our proposal tackles only a subset of traffic problems within a specific institutional setting.

While a future in which transportation looks radically different (e.g., with no human drivers in traffic) is conceivable, and potentially desirable, the current operating environment of AVs is one in which they must interact with human drivers and navigate complex problems of traffic coordination. How people solve such traffic coordination problems, if weighed by the requirements and design principles inspired by virtue ethics, could serve as a blueprint for at least incremental improvements in our day-to-day traffic.

### Case studies

Our proposal would help data curators to dissociate and label wise driving scenarios in order to reorganize and feed them into the algorithm with the purpose of embedding a form of functional driving phronesis into the autonomous system. Of course, our label could also assist computer scientists when they would need to introduce additional filters or solve specific problems. For the remainder of the paper, we will provide two case studies aimed at illustrating the type of driving phronesis that we have argued for.

#### Which lane am I on?

**Fig. 1:**
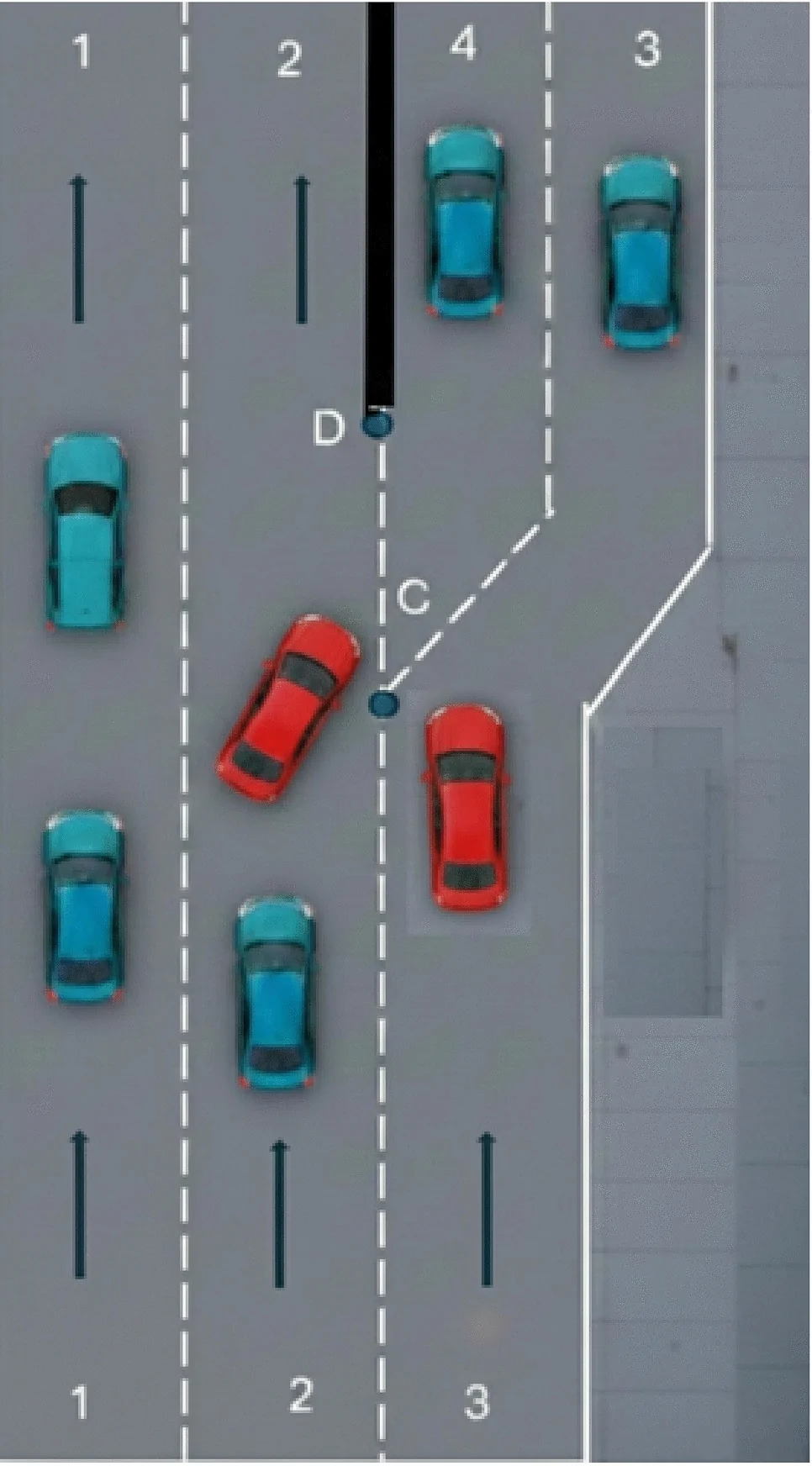
Traffic dilemma with lanes. Source: generated by the authors with Google Gemini based on the drawing of a real case in Bucharest, Romania, coordinates for Google Maps 44.405146, 26.121274

Let us explore the important traffic elements in Fig. 1. The road initially has three main lanes but then expands into four lanes, with lanes 3 and 4 designated for vehicles intending to turn right. During rush hours, lanes 2 and 3 are jammed because most of the traffic faces a bottleneck when cars want to make a right. At point C, most vehicles in lane 2 move into lane 4, while some vehicles in lane 3 either continue straight in their lane or also merge into lane 4.^8^ Most of the time, lane 1 is usually used by cars that go forward to an overpass and after point C, they can also move freely to whichever lane they wish, 1 or 2. At point C, which cars have priority to change direction to lane 4, cars from lane 3 or cars from lane 2? Which of the red cars has priority? Good drivers that want to steer to the right position themselves on lane 3 because this way they do not undercut the queue of the cars from lane 3, some drivers move forward to lane 2 and then change to lane 4. Sometimes good drivers from lane 3 block the road at point C for the cars that come from lane 2 and want to change to lane 4.

A good driver may interpret the behavior of vehicles in lane 2 as a form of free-riding which, in some cases, it indeed is. Although, the cars from lane 2 do not take advantage of the cars from lane 1 because at point C we have no driving rule stating who has priority.

From a consequentialist perspective, one might argue that overall traffic flow is improved when vehicles in lane 3 have access to an additional option, namely lane 4, when preparing to turn right. But there is just more space on lane 2 for cars to queue when they intend to go on the right although they are blocking a lane designated for cars that want to go forward. After several years, the authorities installed a barrier that forces vehicles in lane 3 to continue in their original direction, while allowing vehicles in lane 2 to merge freely into lane 4 (as can be seen at present on Google maps by accessing the coordinates). But this solution might prove ineffective if the traffic flow changes. For example, if traffic toward the overpass intensifies, lane 1 alone may be designated to accommodate the increased flow. This illustrates that apparently simple traffic scenarios can involve a high degree of coordination complexity. Drivers who appear to be behaving badly may, in fact, be following a pattern that proves more efficient and beneficial for overall traffic flow. A good driver must, therefore, be cautious in labeling such behavior as merely selfish or improper, since its value may depend on the broader traffic context. A driver who possesses phronesis can recognize that this situation involves a genuine practical dilemma. Rather than immediately interpreting the behavior of other drivers as simple free-riding, such a driver exhibits a higher degree of tolerance and contextual awareness. They are able to respond prudently, making decisions that do not put themselves or others at unnecessary risk. This form of judgement is not merely passive acceptance, but a practical strategy for achieving legitimate driving goals, such as avoiding accidents, maintaining traffic flow, and arriving at one’s destination on time.

We do not know for sure how an AV might act in similar cases to the one we have discussed, before the authorities installed the fence. If in training the algorithm traffic rules are set as filters, then the AVs could be less effective than human drivers because they will stick to lane 3 all the time even when it is beneficial to be on lane 2 and then switch to 4. The second option is to train the algorithm based on the available data and sort and categorize it in such a way that AVs could be efficient and safe but also be able to recognize when exceptions apply to the rules. In this instance, the people that sort the data could either favor the behavior of a good driver and stick to lane 3, labeling the other as ‘free-riders’, or favor both lanes 2 and 3. But the regulators of transportation systems, alongside the companies that categorize the data to train the models, might benefit from using a framework that encompasses driving phronesis.

#### One lane to rule them all

**Fig. 2:**
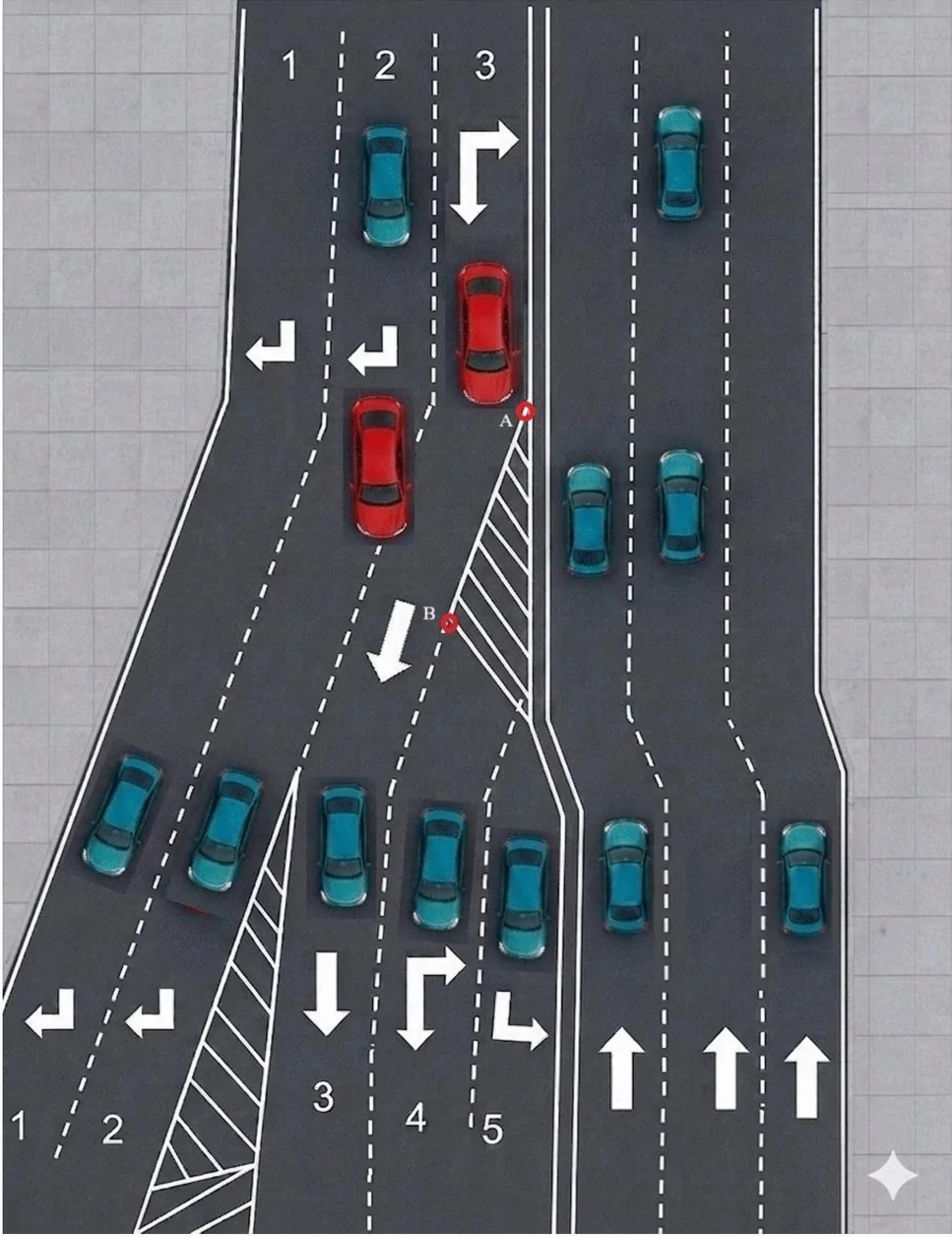
One lane to rule them all. Source: generated by the authors with Google Gemini based on the drawing of a real case in Bucharest, Romania, coordinates for Google Maps 44.415352807698504, 26.10444177970102

Big crowded cities need to come up with all sorts of solutions in order to maximize available space. Traffic authorities designate directions to different lanes to optimize traffic flow, but their success depends on a couple of factors. How much available space do they allow for? How do drivers interpret certain traffic situations? Furthermore, how do they adapt their strategies in order to solve coordination dilemmas like the one in Fig. 2?

We have a main street with two directions. The cars from the left approach the intersection where drivers could continue straight ahead, make a left or a right. Three main lanes labeled in the figure as lane 1, 2, and 3, at point A, inform drivers about which direction is assigned to each lane. Lanes 1 and 2 are assigned to go to the right. Lane 3 is assigned for cars that go straight ahead and which, at point B, will have a 4th lane and later a 5th lane available. The 4th lane is assigned for cars that want to go straight ahead or to the left, while lane 5 is exclusive for cars that want to go to the left. According to traffic rules, the red car on lane 3 has priority, and cars that happened to be on lane 2 need to give way for the cars on lane 3. But most of the time, drivers, quite surprisingly, realize that following the traffic rules for this specific context is not optimal. One lane will be available for two directions, straight ahead and to the left, while the other two, lanes 1 and 2, are assigned for those who make it to the right and do not have to give way to anybody. During rush hour, a queue will be formed because a significant number of cars will try either to position themselves on lane 3 later on, or lane 2 will be blocked by cars that wait for someone to let them change lanes.

There is an informal understanding among drivers that between points A and B cars from lane 3 drive over the painted island straight to lane 4 or, if necessary, to lane 5. Painted islands are useful and very important as buffer zones in other contexts. For example, between lanes 2 and 3, near the intersection, we have another painted island which drivers should not move on or cross because it is dangerous. The cars from lane 2 keep their lane, or switch to lane 3 from point A onwards without much difficulty. This way, wise drivers manage to free up a lane by moving onto the painted island, without putting anyone in danger, and establish an informal convention among themselves. Of course, people breaking this convention do not necessarily lack phronesis, they might lack the local, relevant, context-dependent knowledge needed in order to drive virtuously. Either way, all drivers involved in this mutual understanding need to exhibit a virtuous driving behavior in order to solve the dilemma that arises by the layout of this coordination problem. The fact that people possess the ability to understand rules and contexts, paired with a particular type of knowledge, easily overcomes these sorts of challenges in an optimal way.

## Final remarks

This paper has argued that, when thinking about the place of AVs and the future of mobility and transportation, ethical approaches to AVs design must move beyond the narrow framing of sacrificial dilemmas. While such dilemmas have dominated much of the debate about how AI systems decide in sacrificial dilemma scenarios, they do not capture the ordinary complexity of driving. A more adequate ethics of AVs must, therefore, attend to the nuanced, context-sensitive, and socially embedded forms of judgement required in everyday traffic situations.

Thus, we have argued in favor of expanding the scope of the current debate on AVs by exploring the implications of adopting, within hybrid decision-making methods, elements derived from virtue ethics. This is of high importance especially in the case of end-to-end autonomous driving models, where the training revolves around behavior cloning.

We have introduced the concept of driving phronesis as a cornerstone of understanding how virtuous drivers make decisions in traffic. Furthermore, we have argued that there is a case to be made for training AVs to develop functional driving phronesis, which would help them behave as if they were virtuous and embody virtue, since being genuinely virtuous would entail setting the bar too high.

We consider that the main takeaway of our approach for philosophers, policymakers, engineers and computer scientists active in the AV debate is a useful blueprint for thinking about the milestone that such autonomous systems should reach before being deployed in mixed-traffic environments. Having the functional practical wisdom to know, while being in traffic, what is permissible, how and when paves the way for a design revolution in the world of AVs: they should be conceived, as per our arguments, as avatars of how wise regular median drivers solve coordination problems in everyday scenarios. Or, to link our discussion to an emerging topic in AI ethics, as their DDs. In order to illustrate both the concept of driving phronesis and the challenges involved in our approach, we ended our paper with two examples of non-moral dilemmas which AVs could solve by duplicating the way a phronimos driver behaves.

## Data Availability

No datasets were generated or analysed during the current study.

## Footnotes

1 https://www.sae.org/news/blog/sae-levels-driving-automation-clarity-refinements.

2 https://www.nvidia.com/en-us/solutions/autonomous-vehicles/.

3 Something similar happened in the city of Brașov, Romania, near the end of 2024 when a Tesla car allegedly changed the lane by its own to save the life of a pedestrian, but collided with the car that was moving in the opposite direction: https://www.youtube.com/watch?v=Sxj0Qze-50w.

4 More on virtue ethics in Sect. 3.

5 https://eur-lex.europa.eu/legal-content/EN/ALL/?uri=CELEX:32005L0066.

6 One proposal for keeping humans involved is the “ethical knob”. It allows owners to select settings ranging from how altruistic or egoistic a car should act, to being entirely impartial when the knob is left in the middle (Contissa et al. 2017). Drivers could also have access to other specific elements that influence traffic moral judgments, like driving style, traffic rules compliance, and risk distribution. Distinguishable ethical settings could be identified based on each traffic component (Cecchini and Dubljević 2025). While this adds more complexity, it does not address the deployment of fully autonomous vehicles capable of independent decision-making, since it still keeps humans almost in the driver’s seat.

7 We know little about the third scenario, and remain skeptical regarding the possibility of systems that would coerce humans into driving like artificial agents. For example, “hyper nudges” (Yeung 2016) might eventually alter driving styles if they are designed for the automotive industry, but they are beyond the scope of our paper.

8 At point D lanes 1 and 2 continue their direction onto an overpass and they have no connection with lane 3 and the newly formed lane 4.
